# Social anxiety in adolescence and the first timing of parental home leaving and living with a partner: a longitudinal population-based Young-HUNT3 study in Norway

**DOI:** 10.3389/fpubh.2024.1484501

**Published:** 2024-12-24

**Authors:** Malik Dimbei Halidu, Cathrine Fredriksen Moe, Samira Behboudi-Gandevani, Tommy Haugan

**Affiliations:** ^1^Faculty of Nursing and Health Science, Nord University, Levanger, Norway; ^2^Faculty of Nursing and Health Science, Nord University, Bodø, Norway

**Keywords:** adolescent, social anxiety, leaving a parental home, living with a partner, Young-HUNT3

## Abstract

**Background:**

Social anxiety can make significant life transitions from adolescence to young adulthood particularly stressful. Despite the potential impact, few population-based longitudinal studies have examined the relationship between social anxiety and the timing of key markers of the transition to adulthood. This study investigated the association between social anxiety and the timing of two critical life events: first leaving the parental home and first living with a partner.

**Methods:**

Data were drawn from 8,199 adolescents aged 13–19 who participated in the Young-HUNT3 survey (2006–2008) in Norway, linked with event registration data from Statistics Norway through 2020. Social anxiety was assessed via the Short Form of the Social Phobia and Anxiety Inventory for Children, with scores ranging from 1 to 5. Accelerated failure time (AFT) regression analyses with a Weibull distribution were conducted to evaluate the relationship between social anxiety and the timing of first leaving the parental home and first living with a partner.

**Results:**

The final analytic sample size was 8,045. The median age for first leaving the parental home was 21, whereas the median age for first starting to live with a partner was 24. Higher levels of social anxiety were modestly associated with delays in both life transitions. Specifically, a one-unit increase in the social anxiety score was associated with an approximate one-month delay in leaving the parental home (*T* = 1.005; *p* < 0.05) and a two-month delay in first living with a partner (*T* = 1.010; *p* < 0.001). The predicted mean indicates a difference of 4 months for leaving the parental home and 8 months for first living with a partner, comparing adolescents with the lowest (score of 1) to the highest (score of 5) within the social anxiety spectrum.

**Conclusion:**

This study highlights the association between social anxiety and delays in key life transitions from adolescence to early adulthood. Despite these delays, socially anxious adolescents in Norway appear to reach these milestones—leaving the parental home and living with a partner—in their early twenties, similar to their peers. Although the findings are modest, practically, this information may still hold significant value in informing care providers and policymakers to focus on adolescents as a potential period for implementing evidence-based programs aimed at social anxiety. Future research should explore other stress-inducing life events and examine the long-term socioeconomic and health outcomes of adolescents with social anxiety.

## Introduction

Social anxiety (SA), characterized by an intense and pervasive fear of social situations (1, 2), represents a critical psychological challenge during the transition from adolescence to adulthood (2, 3). Typically, the onset of SA occurs around the age of 15, although it can manifest as early as childhood, even before the age of 10 (4). With a global prevalence of approximately 12% (5), social anxiety disorder (SAD) ranks as the third most common mental health condition worldwide (6). Notably, a recent population-based study in Norway revealed that the prevalence of SA among adolescents aged 13–19 years varies between 2.0 and 5.7%, depending on the diagnostic criteria applied (7).

The transition to adulthood is often marked by significant milestones such as leaving the parental home and entering cohabitation with a partner (8, 9). These milestones are not merely social norms but are also indicative of deeper processes of autonomy and identity formation (8). Demographic research has extensively explored various individual, parental, and contextual factors, including economic resources, housing costs, family dynamics, employment opportunities, and educational trajectories, that influence decisions related to home-leaving and partner living (9–11). However, emerging evidence suggests that psychosocial factors, particularly social anxiety, may also exert a significant influence (12, 13).

Erikson’s psychosocial development theory (14) provides a theoretical lens to understand the complexities of the journey from adolescence to young adulthood. This model emphasizes the pivotal tasks of identity formation; establishing independence and self-sufficiency is normative, which involves individuating from one’s family and establishing intimate relationships during this transitional phase (15). As adolescents grapple with these challenges, Erikson’s framework becomes instrumental in deciphering the psychosocial dynamics shaping their developmental trajectory and underscores adolescence as a critical period for forming autonomy and identity (14, 15). Erik Erikson posited that during adolescence, individuals face the challenge of forming a coherent, integrated, and stable self-identity; failure to achieve this may complicate the transition into adult roles and responsibilities (16). The onset of SA during mid-adolescence may disrupt this developmental trajectory, particularly in areas such as relationship formation and autonomy seeking (17–21). These disruptions are not transient; they may persist into adulthood, potentially contributing to the long-term psychosocial challenges observed in individuals with early-onset social anxiety (13, 14).

Despite the importance of adolescents’ identity formation in the extant literature, there remains a notable gap concerning the relationship between social anxiety and the timing of critical life transitions, such as leaving the parental home or beginning to live with a partner. While studies suggest that identity achievement is often incomplete by the end of high school and continues to evolve into the twenties (22–25), few studies have explored how social anxiety might influence these transitions. This gap is particularly striking given the potential lifelong implications of social anxiety for personal development and social functioning (25).

Norway provides a context for examining these issues. The country’s high standard of living and progressive social policies offer a supportive environment for adolescents navigating the transition to adulthood. Norwegian culture, characterized by values of equality, independence, and acceptance of diverse family structures—including cohabitation—fosters an environment where nearly half (44%) of individuals aged 20–29 live independently, and 51% live with a partner (26–28). This demographic landscape is distinctive within the Organization for Economic Cooperation and Development. Erikson’s concept of identity development assumes opportunities for exploration and choice, which may vary across global contexts (9, 29). However, it has utility in the Norwegian context, where societal structures and dominant values such as independence and individuality encourage identity exploration and, therefore, offer an opportunity to explore the associations between social anxiety and critical life events.

Accordingly, the primary research question investigates how social anxiety influences the timing of two crucial life transitions: first, leaving the parental home, and first, living with a partner. These inquiries are grounded in Erikson’s theory, which postulates that social anxiety may hinder identity consolidation and the process of achieving autonomy and intimacy. Our hypotheses propose that higher levels of social anxiety are associated with delayed life transitions, aligning with broader perspectives on psychosocial development.

*H1*: Elevated social anxiety will be associated with a delayed timing of first leaving the parental home, reflecting the challenges of negotiating autonomy and connection during the transition to adulthood.

*H2*: Elevated social anxiety will be linked to delayed initiation of first cohabitation with a partner, highlighting the potential impact of social anxiety on forming intimate relationships during emerging adulthood.

Therefore, this study aims to assess whether social anxiety is associated with (a) the timing of first leaving parental home and (b) the timing of first living with a partner.

## Methods

### Data source and study population

This study leverages data from the Young-HUNT3 survey (2006–2008), a sub-study of the third Trøndelag Health Study (HUNT) (30). The HUNT study, a large-scale, cross-sectional, population-based health survey conducted in Trøndelag County, Norway, offers a robust dataset for examining adolescent health (31). Previous research has extensively documented detailed descriptions of the HUNT study and its adolescent component, the Young-HUNT (32, 33).

In the Young-HUNT3 survey, all adolescents aged 13–19 years residing in the former Nord Trøndelag County were invited to participate, comprising 10,464 potential respondents. Data collection was primarily conducted via a self-report questionnaire administered during school hours in lower secondary (ages 13–16) and upper secondary schools (ages 16–19). Adolescents not enrolled in school received the questionnaire by mail (31). The survey achieved a participation rate of 78.4%, with 8,199 adolescents completing the questionnaire (32). To enrich the dataset, Young-HUNT3 data were linked with several national registries, including the Statistics Norway (SSB) population database, the Norwegian National Education Database (NUDB), and the Central Population Register (CPR) (34, 35). This integration, facilitated by the unique 11-digit national personal identification number assigned to each Norwegian citizen, resulted in a final analytic sample of 8,045 participants. The longitudinal data spans from 2005–2020, providing a comprehensive view of the transition from adolescence to young adulthood in Norway.

### Measures

#### Outcomes of the study

The primary outcomes of interest were the time-to-event for two key life transitions during emerging adulthood: (1) leaving the parental home and (2) first living with a partner. Binary status variables were created for each transition, tracking occurrences between ages 13 and 34, based on 19 items from SSB records about registered family types at each time point (i.e., in the fourth quarter of each calendar year from 2005 to 2020). Once a participant experienced an event, they were excluded from further data collection for that event (coded as missing). Events occurring before age 16 were recorded as not having occurred (see Appendix 1 for details).

#### Exposure variable

Social anxiety was assessed via a nonclinical symptom index derived from the Social Phobia and Anxiety Inventory for Children (SPAI-C) short form, which is included in the Young-HUNT3 questionnaire. The inventory consists of six items on a Likert scale (1 = never to 5 = always), capturing self-reported experiences of anxiety in social contexts. Sample items include “I feel anxious and do not know what to do in an embarrassing situation” and “I feel anxious when I am with others and have to do something while they watch me do it (e.g., be in a play, play music, play sports).” The composite score, calculated as the mean of the item scores, ranges from 1 to 5, with higher scores indicating greater perceived social anxiety. The scale’s Cronbach’s alpha of 0.85 suggests high internal consistency.

#### Control variables

We included several control variables from the Young-HUNT3 survey and SSB data to mitigate potential confounding effects. These variables include gender, family structure, conflict, income, and educational status at age 25. Family structure was classified based on whether parents had been together or apart for at least 1 year. Family income was measured by the participants’ subjective assessment of their family’s financial well-being. Educational status at age 25, which often coincides with the completion of tertiary education, was also considered. Family conflict was categorized into two groups based on the response to whether conflicts occurred: (1) no conflict and (2) yes conflict (within the last 12 months or before).

#### Analytical procedure

Descriptive statistics of frequencies, proportions, means, and standard deviations (SDs) were used to summarize the participants’ characteristics. Kaplan–Meier survival analysis was employed to estimate the mean survival age for leaving the parental home and first living with a partner. Further analysis explored the relationships between covariates and these outcomes based on the mean survival age.

Given that the proportional hazards assumption was violated (as indicated by analyses of Schoenfeld residuals; see Appendix 1), we applied accelerated failure time (AFT) models. AFT models are particularly advantageous for time-to-event data analysis, allowing for censored data and not relying on the proportional hazards assumption, making them suitable for our study’s objectives (36). Two hierarchical models were constructed for each outcome variable. Model 1 included only social anxiety and the outcome variables, whereas Model 2 was adjusted for potential confounders. To ensure that unobserved school-level characteristics did not confound the results (e.g., differences in social anxiety levels due to varying school environments), we explored potential effect modification at the school level (i.e., lower secondary vs. upper secondary) and allowed parameters to vary across these levels, as determined by a likelihood ratio test.

To address issues of truncation and censoring, the survival characteristics of the dataset were defined to include the year of study entry (2006–2008), event or censoring status, date of birth, and year of event observation. We compared the fit of different probability distributions via information criteria (AIC and BIC) and selected the Weibull distribution as the best fit for both outcomes (see Appendix 2). All the statistical analyses were conducted via STATA (version 17; STATA Inc., College Station, TX, USA), with the significance level set at *p* < 0.05 and 95% confidence intervals. Marginal plots were generated to visualize the associations between social anxiety and the outcomes of interest.

A sensitivity analysis was performed for robustness, estimating unordered failure events for different types and accounting for the correlation between leaving the parental home and living with a partner. We allowed the baseline hazard function to vary by outcome type, achieved by categorizing the data based on the outcome type, enabling each category to have its baseline hazard function while maintaining consistent coefficients across categories (37).

#### Missing data

We conducted a missing completely at random (MCAR) analysis to assess the impact of missing data. Little’s MCAR test indicated no significant variation between the means of different missing-value patterns for social anxiety and the outcome variables of interest: first living with a partner (𝛸^2^ (0.66) = 1, *𝑝* = 0.42) and moving out of the parental home (𝛸^2^ (0.46) = 1, *𝑝* = 0.50). Therefore, missing data for the main variables of interest were classified as MCAR. Given that missing data occurs at the item level in the baseline survey, rather than from loss to follow-up over time, and that the GSEM employs maximum likelihood estimation, our parameter estimates are efficient and consistent under the assumption that data are missing at random, and the model is correctly specified. This approach enhances the robustness of our findings with respect to missing data.

## Results

The baseline characteristics of the study participants are outlined in Table 1. Among the 8,045 adolescents included in the study, 50.4% were female. At the outset (2006–2008), 136 participants (3.73%) had already left their parental home, and eight (0.12%) were living with a partner. By 2020, the vast majority—7,891 participants (98.1%)—had moved out of their parental homes, and 7,080 (88%) were living with a partner. The average age of the participants at baseline was 15.9 years (SD = 1.7). The youngest participants at entry were 13 years old, whereas the oldest observed ages for leaving home and starting to live with a partner in 2020 were 32 and 34 years, respectively. The Kaplan–Meier estimates revealed median ages of 21 years for those who left the parental home and 24 years for those who started living with a partner (Supplementary Figures 1A,B).

**Table 1:** Descriptive statistics for the Young-HUNT3 data and SSB variables.

Variable	Observation	Mean (SD)	% Missing
Self-reported variables from Young-HUNT3			
Social anxiety score	7,846	1.9 (0.72)	2.47
Sex (*n*, %)			
Female	4,055 (50%)	–	0.0
Male	3,990 (49.6)	–	
Parents living status	7,486		6.95
Living Together	5,105 (68%)	–	
Not living together	2,381 (31.8%)	–	
Parents financial status			
Middle (equal)	5,431 (67.5%)	–	
Well-off (Better)	1,358 (16.9%)	–	
Poor (Bad)	699 (8.7%)	–	6.9
Family conflicts			
No	4,428 (55%)		
Yes	3,081 (38%)		7
Education level			
Upper secondary school	3,278 (40.8)		
Lower secondary school	4,658 (57.9%)		1.35
Based on administrative information from SSB and Young-HUNT3			
Leaving parents’ home			
Age at baseline (Study Entry)	8,045	15.9 (1.78)	0.00
Age at leaving parent home (Exit)	8,045	21.3 (2.7)	0.00
Living with first partner			
Age at baseline (Study Entry)	8,045	15.9 (1.78)	
Age at leaving parent home (Exit)	8,045	24.80 (3.0)	
Current education level@25			
Not in tertiary	4,163 (51.8%)	–	
In tertiary	3,882 (48.3%)	–	

Table 2 presents the observed means for each unit increase in the social anxiety score, showing an average delay of approximately 1 month for leaving the parental home and 2 months for first living with a partner. These findings underscore the subtle yet measurable association of social anxiety with the timing of these significant life events.

**Table 2:** Observed means for social anxiety score and time to event of first leaving parents’ home and living with first partner life events.

Social anxiety score	Observed mean for first leaving parents’ home	Observed mean for living with a partner
1	21.87	24.60
2	21.99	24.80
3	22.12	25.07
4	22.25	25.34
5	22.28	25.45

### Survival models

Table 3 details the results of the survival models for the time to first leave the parental home and first live with a partner. The unadjusted time-to-event model for leaving the parental home revealed that higher levels of social anxiety were associated with a decrease in the accelerated failure rate for the event (*T* = 1.005, *p* < 0.01). Specifically, a unit increase in the social anxiety score was linked to a 5% increase in the mean survival time, indicating a deceleration in the time-to-event. Supplementary Figure 2 illustrates the predicted mean survival time, showing that higher social anxiety scores correspond to longer delays in leaving the parental home.

**Table 3:** Estimated time ratios (standard errors) of the AFT MODEL.

	Time to first leaving – out of parents’ home						Time to First living with a Partner					
	Unadjusted model		Adjusted model				Unadjusted model			Adjusted Model		
	Time ratio	*p*-values	Time ratio	*p*-values	Time ratio	*p-* values	Time ratio	*p*-values	Time ratios	*p*-values	Time ratios	*p*-values
			USS		LSS				USS		LSS	
Covariates												
Social Anxiety Index	1.005* (0.002)	0.021	0.997 (0.003)	0.34	1.002 (0.003)	0.532	1.010*** (0.002)	0.000	1.002 (0.003)	0.491	1.010** (0.003)	0.002
Gender												
Reference (female)												
Male			1.002 (0.004)	0.677	0.997 (0.003)	0.358			1.008 (0.005)	0.074	0.993 (0.004)	0.068
Parents living status												
Reference (living together)												
Not living together			0.997 (0.004)	0.504	1.000 (0.004)	0.913			0.999 (0.005)	0.792	0.997 (0.005)	0.526
Family conflicts												
Reference, (NO)												
Yes			0.995 (0.004)	0.182	1.000 (0.004)	0.925			0.0.994 (0.005)	0.217	1.001 (0.004)	0.805
Educational status at age 25												
Reference (not in tertiary)												
In tertiary			1.001 (0.004)	0.743	1.013*** (0.003)	0.000			0.995 (0.005)	0.303	1.018*** (0.004)	0.000
Family finance												
Reference (MI)												
Well – off			0.0.998 (0.005)	0.707	0.994 (0.005)	0.149			1.002 (0.006)	0.699	1.003 (0.006)	0.643
Poor			0.994 (0.007)	0.353	1.001 (0.006)	0.921			1.014 (0.008)	0.087	0.0.987 (0.007)	0.079
*N*	7,838		2,956	4,117					7,838	3,055	4,132	

MI, Middle Income; LSS, Lower secondary school participants; USS, Upper secondary school participants; standard errors in parenthesis, Bold values indicate statistically significant results; ***p < 0.001, **p < 0.01, *p < 0.05.

Similarly, the unadjusted time–to–event model for starting to live with a first partner indicated that greater social anxiety was significantly associated with a decrease in the accelerated failure rate for this event (*T* = 1.010, *p* < 001). A unit increase in the social anxiety score resulted in an approximately 10% increase in the mean survival time, further delaying the event. Supplementary Figure 3 shows an increase of approximately 2 months in the mean survival time before starting to live with a partner for each unit increase in the social anxiety score. The adjusted model revealed statistically significant differences for adolescents in lower secondary school (*T* = 1.010, *p* < 0.01) but not for those in upper secondary school (*T* = 1.002, *p* > 0.05).

### Sensitivity analysis

Stratified Cox regression via the Efron method confirmed the robustness of these findings. The results showed consistent patterns in both the unadjusted model (*T* = −0.06, *p* < 0.05) and the adjusted model, controlling for education, gender, family income, family structure, and family conflicts (*T* = −0.06, *p* < 0.05).

## Discussion

The journey from adolescence to adulthood, marked by leaving the parental home and navigating changes in relationship status, is a complex process extensively explored in youth transition research (8, 9, 38). Over the last century, this transition has seen both postponement and diversification, reflecting an increasingly individualized life trajectory (39, 40). Our study investigated the relationship between social anxiety and the timing of pivotal life transitions—first, leaving the parental home and first living with a partner. Using a Norwegian population-based dataset covering the calendar years 2005–2020 and applying survival analysis, we provide new insights into the underexplored area of elevated social anxiety in adolescents and its association with the timing of significant milestones in Norway.

This long-term follow-up study revealed that half of the participants left their parental home or initiated their first living arrangement with a partner at the ages of 21 and 24. These trends are consistent with similar figures observed in Luxembourg and the Nordic countries Sweden, Denmark, and Finland, where the average age for leaving parental homes is less than 22 years (41), and cohabitation typically occurs between ages 25 and 29 (42). In contrast, in countries such as Croatia, Italy, and Bulgaria, people tend to leave their family home or live with a partner at an average age greater than 30 years (43).

Previous research has linked anxiety, conduct disorders, and antisocial behavior in youth with partnership difficulties in adulthood, including inter-partner conflict, violence, and abuse (16–18, 44–46), as well as delayed patterns of forming relationships (47). While earlier studies have primarily focused on these critical life events in adolescence or adulthood, this study is, to the best of our knowledge, the first to examine social anxiety and the transition from adolescence to young adulthood. Our findings reveal statistically significant associations between social anxiety and the timing of both leaving the parental home and initiating a first living with a partner. However, the results suggest that a one-unit increase in social anxiety correlates with an approximately one-month delay in leaving the parental home and a two-month delay in living with a first partner in the unadjusted model. While we did not find any statistically significant difference based on school level at the time of study entry for adolescents leaving their parents’ home, in the case of those living with a partner, there was a statistically significant difference for those in lower secondary school but not in upper secondary school.

The modest differences between the observed and predicted mean ages at these outcome events may be partly due to Norwegian cultural, economic, and sociodemographic factors. In Norway, young people may feel a growing expectation from friends and family to leave their parental home after completing upper secondary school, typically at the age of 19. The first cohabitation with a partner also often occurs before marriage and the establishment of a binding family. Individuals with social anxiety strive to follow these social norms to avoid being perceived as different and inadequate. In other cultures where living with one’s parents in adulthood is more common and cohabitation without marriage is less typical, social anxiety may have a more significant effect on these life choices during the transition from adolescence to adulthood. In addition, we might have found more pronounced differences if we had clinical data, including diagnoses of social anxiety disorder (SAD), although adolescents with SAD may be less likely to seek clinical help because of the fear of evaluation, which could introduce biases into clinical samples (6).

Our modest estimation within Erikson’s psychosocial theory (14, 15) offers a theoretical lens for understanding identity development from adolescence to young adulthood. Erikson’s psychosocial theory is a developmental framework that outlines a series of stages individuals go through across the lifespan, each marked by a unique psychosocial crisis or challenge. According to Erikson, successful resolution of these crises contributes to healthy psychosocial development, whereas failure to navigate them can result in difficulties in later stages.

The stage particularly relevant to the transition from adolescence to young adulthood, specifically the process of moving out of parental homes or first living with a partner, is Erikson’s “Intimacy vs. Isolation” stage (15). This stage occurs during young adulthood, between approximately 19 and 40 years (48). In this stage, individuals face a psychosocial crisis of intimacy versus isolation (15, 48). Successfully resolving this crisis involves the development of meaningful and intimate relationships while also establishing a degree of autonomy.

Taken together, our findings, though modest, show that these differences are meaningful in a broader developmental and social context. Delays in reaching key milestones, even if brief, may signal underlying social or emotional challenges associated with social anxiety. For some individuals, these delays could reflect hesitation in seeking independence or forming relationships, potentially leading to cumulative effects on their confidence and social integration during adolescence to young adulthood. This pattern suggests an opportunity for early support to help socially anxious individuals navigate these transitions more smoothly. While we recognize that the delays are not extensive, they may still accumulate disadvantages, limiting some individuals’ freedom to lead a life they value. We suggest developing public health policies and initiatives designed for universal implementation among adolescents but proportionally directed toward socially anxious individuals and sub-groups based on their needs. In addition, the study suggests a focus on evidence-based approaches and investments in skills development, such as social–emotional learning at school and home, to equip adolescents with tools for stress management.

The study contributes to the literature by employing a robust methodology that combines survey data (Young-HUNT3) with national administrative records, resulting in a comprehensive and accurate dataset that minimizes recall bias and enhances data quality. This integration is a major strength, as it enhances the external validity of our results by leveraging a sizable population-based cohort and objective outcome measures, thereby strengthening the reliability and validity of our findings. Additionally, the longitudinal design allows for the tracking of participants over time, which is essential for examining the relationship between social anxiety and key life milestones, such as leaving the parental home and cohabiting with a partner.

However, it is essential to interpret our findings with caution due to the limitations inherent in our study. The correlational nature of the study limits the ability to make causal assertions. Additionally, the study uses annual-level data with no details of specific months or seasons within the year. This suggests that any observed or censored event is tied to yearly intervals, which can obscure whether the event happened at the beginning or end of the year, which might lead to overestimation or underestimation because individual events at the start or end of the year are not distinguished. This can bias survival estimates, as yearly data might make transitions seem longer or shorter than they are. Furthermore, there are challenges related to residential relocations and cohabitation initiation times, which may affect the accuracy of reported shared living arrangements. Misclassification may occur because some individuals might not report their move according to the National Central Population Register (CPR) regulations, which state that a person must be registered as a resident where they primarily spend their daily rest. This issue is most pronounced among students enrolled in higher education. However, since January 1, 2014, Statistics Norway has implemented measures, including data sources and statistical methods, to better determine and improve the establishment of households for students with a de jure address at their parents’ place of residence. Substitute addresses have been collected from sources other than the CPR for students. Thus, at an individual level, there are some potential discrepancies between registered residential relocations and times of cohabitation initiation and the actual commencement of shared living arrangements. Moreover, the temporal constraint of a single-time-point measurement of the social anxiety scale potentially overlooks fluctuations or patterns that might emerge in longitudinal data which necessitates caution in interpreting the observed associations. Also, self-reported data (Young-HUNT3) can introduce biases such as social desirability, which may skew the accuracy of participants’ responses. Another important limitation of the Young-HUNT3 study was that it was completed at school, introducing challenges related to school attendance that may influence outcome scores. Furthermore, this study did not evaluate the potential influence of Norwegian culture—where young people may face increasing expectations from friends and family to move out of the parental home upon completing upper secondary school, typically around age 19— (49). Lastly, while including interaction models and multilevel approaches could have provided deeper insights into the complexity of the relationship between social anxiety and key life transitions in adulthood, this was not possible, given the limitations of the available data.

## Conclusion

In conclusion, our study demonstrated the association between social anxiety and delays in key life transitions, including leaving the parental home and living with one’s first partner, as critical milestones during adolescence to young adulthood. Importantly, these findings suggest that although social anxiety may delay these transitions, most individuals with elevated anxiety levels eventually achieve these transitions, albeit slightly later than their peers. While the delays observed were modest, this information may still hold significant value in informing care providers and policymakers to focus on adolescents as a potential period for implementing evidence-based programs aimed at social anxiety. This insight may be particularly relevant in the context of Norwegian culture, where societal expectations may buffer against the more severe consequences of social anxiety observed in other cultural contexts.

Future research with a larger sample size is warranted to investigate the broader implications of social anxiety for other life events and choices, particularly across different sociocultural contexts. Studies utilizing repeated measures of social anxiety, along with other psychological factors, would provide a more detailed understanding of how these factors evolve and interact over time, influencing the trajectory of young adults through complex life transitions. Additionally, including of clinical assessments of social anxiety could further clarify its effects across different population groups. Furthermore, examining interactions between social anxiety and contextual factors, such as socioeconomic status, family structure, and school environment, would provide a more comprehensive view of the conditions under which social anxiety influences key adulthood milestones.

## Data Availability

The datasets presented in this article are not readily available because given the legislation governing the use of the HUNT dataset and population registry data provided by Statistics Norway, our data cannot be publicly available. However, the data supporting this study’s findings are available on request per the agreement with the owner of the data, HUNT Research Centre and Statistic Norway, and the approver of the research, the Regional Committees for Medical and Health Research Ethics (REC). Requests to access the datasets should be directed to kontakt@hunt.ntnu.no.
