# Supplementary material for: Social anxiety in adolescence and the first timing of parental home leaving and living with a partner: a longitudinal population-based Young-HUNT3 study in Norway
Source: Front Public Health. 2024 Dec 24;12:1484501. doi: 10.3389/fpubh.2024.1484501 (PMC11703836; doi:10.3389/fpubh.2024.1484501)
Supplement: Supplementary file 1 [file Table_1.docx]

Supplementary Material

# Supplementary Data

**Appendix 1**: Two transition tasks in emerging adulthood were used to model outcomes: (a) leaving 'parents' home and (b)initiating time to first living with a partner(i.e., marriage/cohabiting partner). Home leaving and first living with a partner were defined based on 19 items from the family type registration (i.e., 3-digit code) records from SSB. The items are; 1-One-person family, person under 30 years of age (code-111), 2. One-person family, person 30-44 years old (code-112), 3. Married couple with small children, youngest child 0-5 years(code-211), 4. Cohabiting couple with small children- youngest child 0-5 years(code-212), 5. Married couple with large children -youngest child 6-17 years(code-221), 6. Cohabiting couple with older children -youngest child 6-17 years old (code-222), 7. Mother with small children -youngest child 0-5 years(code-231), 8. Father with small children -youngest child 0-5 years(code-232), 9. Mother with large children -youngest child 6-17 years(code-241), 10. Father with grown children, youngest child 6-17 years(code-242), 11. A married couple without children, the oldest person under 30 years of age (code-311), 12. Married couple without children, oldest person 30-44 years(code-312), 13. Cohabiting couple without children, oldest person under 30(code-315), 14. Cohabiting couple without children, oldest person 30-44 years(code-316), 15. Cohabiting couple without children, oldest person 45-66 years(code-317), 16. A married couple with adult children, youngest child 18 years and over(code-321), 17. Cohabiting couple with adult children, youngest child 18 years and over(code- 322), 18. Mother with adult children, youngest child 18 years and over(code-331) and 19. Father with adult children, youngest child 18 years and over(code-332). Leaving 'parents' home was defined based on 12 items (i.e., 3,4,5,6,7,8,9,10,16,17,18,19). Regarding living with a partner, seven items were utilized (i.e., 1,2, 11,12,13,14 and 15). For each age from 13 –34, a binary variable for each status was created indicating whether the individual occupied the status for the first time at that age (coded 1), or had not occupied the status by that age (coded 0). Once the individual occupied one of the role statutes, they no longer contributed data for the remaining ages for that status (coded as missing). Events that occurred before the age of 16 was recorded as not occurred.

# Supplementary Figures and Tables

**APPENDIX 2: Test of proportional-hazards assumption**

|  | rho | | chi2 | | df | | Prob>chi2 |
| --- | --- | --- | --- | --- | --- | --- | --- |
| Gender | -0.018 | | 1.990 | | 1 | | 0.158 |
| Parents relate. status | 0.006 | | 0.220 | | 1 | | 0.642 |
| Social anxiety | 0.002 | | 0.020 | | 1 | | 0.897 |
| Current Education | 0.010 | | 0.630 | | 1 | | 0.429 |
| Parents financial status | -0.009 | | 0.520 | | 1 | | 0.469 |
| Global test | | 395.240 6 | |  | | 0.000 | |
|  | | | | | | | |

**APPENDIX 3: Goodness of fit statistics**

| Model | ll(model) | df | AIC | BIC |
| --- | --- | --- | --- | --- |
| Lognormal | -18710.92 | 8 | 37437.840 | 37493.010 |
| Weibull | -16920.36 | 9 | 33858.710 | 33920.770 |
| Loglogistic | -18274.19 | 9 | 36566.380 | 36628.440 |

## Supplementary Figures

Figure 1: Survival estimates (in years)for (A) moving -out of ' 'parent's home and (B) for first living with a partner.

[A]

[B]


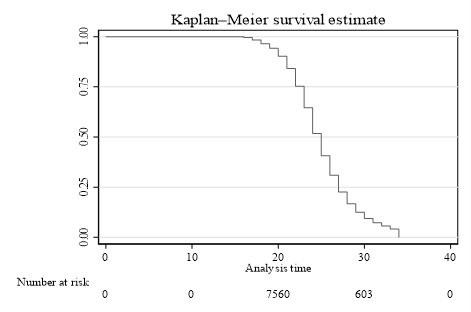


Figure 2: Predicted marginal mean survival time to leave 'parents' home by social anxiety level

Figure 3: Predicted marginal mean survival time-to-first living with a partner by social anxiety level
